# A Chalcone Glycoside from the Fruits of *Sorbus commixta* Hedl.

**DOI:** 10.3390/molecules14125323

**Published:** 2009-12-16

**Authors:** Lok Ranjan Bhatt, Moon Sung Bae, Bo Mi Kim, Gi-Su Oh, Kyu Yun Chai

**Affiliations:** 1 Department of Bionanochemistry, School of Natural Sciences, Wonkwang University, Iksan, Chonbuk 570-749, Korea; E-Mails: lokranjan2000@yahoo.com (L.R.B.); snowcat@wonkwang.ac.kr (M.S.B.); 123456@wonkwang.ac.kr (B.M.K.); 2 Vestibulocochlear Research Center, School of Medicine, Wonkwang University, Iksan, Chonbuk 570-749, Korea; E-Mail: echemogs@wku.ac.kr (G.-S.O.)

**Keywords:** *Sorbus commixta* Hedl., rosaceae, chalcone glycoside, neosakuranin

## Abstract

*Sorbus commixta* Hedl. (Rosaceae) has been traditionally used in oriental countries for the treatment of asthma and other bronchial disorders. In this study, a chalcone glycoside was isolated from the ethyl acetate extract of the fruits of this plant. The compound was identified as neosakuranin based on the spectroscopic analysis and comparion with literature data. This is the first report of isolation of neosakuranin from *Sorbus commixta.*

## Introduction

*Sorbus commixta* Hedl. (Rosaceae) has been used for the treatment of cough, asthma, and other bronchial disorders in East Asian countries, including Korea, China and Japan [1,2,3,4,5]. The plant is reported to have promising antioxidative [2], anti-atherogenic [3], anti-inflammatory [1], anti-atherosclerotics [4] and vascular relaxant effects [5], and it also reduces hepatic lipid peroxidation by decreasing the bioavailability of alcohol and its oxidative metabolites through the protection of hepatic catalase [6]. Although, several papers have been published on the pharmacological properties of crude extracts [1,2,3,4,5,6], only a few studies have been carried out on the phytochemical composition of *S. commixta.* Previous works on the plant had led to the isolation of prunasin and amygdalin [7] and some triterpenoids such as lupenone and lupeol [8,9]. This paper deals with the isolation of a chalcone glycoside from the fruits of *S. commixta.*

## Results and Discussion

Fractionation and purification of the methanol extract of *S. commixta* fruits using silica gel and Sephadex LH-20 column chromatography together with preparative high performance liquid chromatography (HPLC) led to the isolation of the chalcone glycoside **1**. The compound was identified by the assignment of the ^1^H- and ^13^C-nuclear magnetic resonance (NMR), heteronuclear multiple bond connectivity (HMBC), heteronuclear multiple quantum coherence (HMQC), correlated spectroscopy (COSY) together with mass spectrum (MS), infrared (IR), ultraviolet visible (UV-vis) spectra and comparison with the literature data [10,11,12]. The ^1^H-NMR spectrum of **1** showed a pair of *meta*-coupled aromatic protons at *δ* 6.34 and 6.15 (1H each, both s, 3’, 5’-H), *ortho*-coupled A_2_B_2_ type aromatic protons at *δ* 6.84, 7.62 (2H each, both d, *J* = 7.8 Hz; 3, 5 and 2, 6-H), and a pair of *trans*-olefinic protons at *δ* 8.01, 7.70 (1H each, both d, *J* = 15.1 Hz; *α, β*-H) together with one glucopyranosyl part (*δ* 5.19, d, *J* = 7.3 Hz; 3.48-3.54, m; 3.39 - 3.45, m; 3.72, dd, *J =* 5.5 and 11.9 Hz; 3.91, d, *J* = 11.9 Hz) and aromatic methoxyl group (*δ* 3.83, s, 3H).

**Figure 1:**
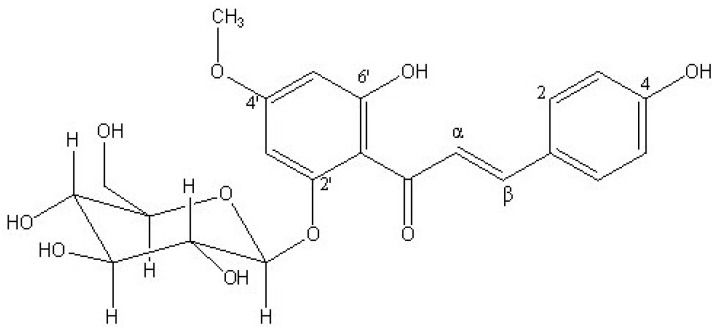
Structure of compound **1**.

The characteristic *trans*-olefinic protons at *δ* 7.70, 8.01 (both d, *J =* 15.1 Hz) and the ^13^C-NMR signal at *δ* 193.43 (C=O) suggested that compound **1** was a chalcone [10,11]. The connectivity of glucose moiety and methoxyl group was determined based on HMBC and COSY experiments. In the HMBC spectrum of **1**, correlation of *O*-methyl protons (*δ* 3.83, s), H-3’ (*δ* 6.34, s), and H-5’ (*δ* 6.15, s) with C-4’ (*δ* 165.87) was observed and the COSY spectrum showed correlation between H-3’ (*δ* 6.34, s), and H-5’ (*δ* 6.15, s), suggesting the location of the *O*-methyl group at the C-4’ position. HMBC correlation of the anomeric proton (*δ* 5.19, d, *J* = 7.3 Hz, H-1") with the carbon at δ 160.06 (C-2’) indicated the location of glycoside group at C-2’ position [10,11]. Moreover, the large coupling constant (*J* = 7.3 Hz) of the anomeric proton at *δ* 5.19 indicated the presence of the *β*-glucose moiety. Based on above spectroscopic data and comparison with the literature [11,12], the compound was identified as neosakuranin (Figure 1). This is the first report of neosakuranin from *Sorbus commixta*.

## Experimental

### General

Nuclear magnetic resonance (NMR) spectra were recorded in CD_3_OD on a JEOL JNM-ECP 500 spectrometer (operating at 500 Hz for ^1^H-NMR and 125 Hz for ^13^C-NMR). The mass spectrum was recorded on a LC-MCD Trap 00099. The UV-vis spectrum was recorded on a Hewlett Packard UV-Vis Diode Array Spectrophotometer. The IR spectrum was measured on a Prestige-21 FTIR spectrometer (Shimadzu). TLC was carried out on precoated Silica gel F_254_ plates (Merck, art. 5715) and spots were detected under UV (254 and 366 nm). Column chromatography was performed using Silica gel 60 (Merck, 40–63 and 63–200 μm) and Sephadex LH-20 (Sigma, Amersham Biosciences, Ltd, 25–100 μm). HPLC analysis was carried out using a Sykam liquid chromatograph equipped with a Sykam S 3240 UV detector, C-18 column (Daiso, 120 ODS, 250 mm × 20 mm 5 μm), an injection valve with a 500-μL loop and Peak Simple software, model 202/203 (SRI instruments, USA). 

### Plant material

*Sorbus commixta* fruits were purchased from a herbal store in Iksan, Korea in September 2008. The plant material was identified by Prof. Choi Han Gil, Department of Biology, Wonkwang University. A voucher specimen (SC-0057) has been deposited at the Herbarium of the Department of Biology, Wonkwang University, Korea.

### Extraction and isolation

*Sorbus commixta* fruits (1.2 kg) were extracted with methanol (4 L) at room temperature for three days. The solvent was evaporated under reduced pressure to yield the MeOH extract (25.5 g). The MeOH extract was suspended in H_2_O and was extracted successively with *n*-hexane, EtOAc, and *n*-BuOH to furnish the corresponding *n*-hexane (4.25 g), EtOAc (6.5 g), and *n*-BuOH (8.4 g) extracts and an aqueous residue. A part of the EtOAc extract (4.0 g) was subjected to silica gel (120 g) column chromatography eluting with the mixtures of CH_2_Cl_2,_ EtOAc and MeOH (9:1:0 - 0:1:1). Fifty mL fractions were collected and combined into six major fractions based on TLC. Fraction 3 (450 mg; collected from 9:1–7:3; EtOAc: MeOH) was submitted to Sephadex LH 20 (25 g) column using *n*-hexane-CHCl_3_- MeOH (1:1:1) as eluent and collected in 24 subfractions of 25 mL each. Subfractions 10-15 (110 mg) were combined and submitted to prep HPLC (Waters 120 ODS-BP, 250 mm × 20 mm, 5 μm), eluted with acetonitrile (A) and 0.2% acetic acid in H_2_O (B) (0 min 10% A, 55 min 55% A, 3 min 100% A and 10 min 10% A) at a flow rate of 3 mL/min to give compound **1** (15 mg, tR 64 min). Pale yellow gum, UV_max_ (MeOH) 262 and 371 nm, IR (KBr) cm^-1^: 825, 1095, 1168, 1219, 1280, 1346, 1460, 1544, 1562, 1631, 1653, 2924, 3448; ESI-MS *m*/*z* 472.4 [M+Na]^+^ (calcd. 471.4 for C_22_H_24_O_10_Na); ^1^H and ^13^C-NMR data for **1**. ^1^H-NMR: *δ* 8.01 (1H, d, *J* = 15.1, H-*α*), 7.70 (1H, d, *J* = 15.1, H-*β*), 7.62 (2H, d, *J* = 7.8, H-2,6), 6.84 (2H, d, *J* = 7.8, H-3,5), 6.34 (1H, s, H-3’), 6.15 (1H, s, H-5’), 5.19 (1H, d, *J* =7.3, H-1"), 3.39 - 3.45 and 3.48-3.54 (4H, m, H-2",3",4",5"), 3.72 (1H, dd, *J* = 5.5 and 11.9 Hz, H-6" ), 3.91 (1H, *J* = 11.9 Hz, H-6"), 3.83 (3H, s, OMe); ^13^C-NMR: *δ* 124.45 (C-*α*), 143.31 (C-*β*), 193.43 (C=O), 127.11 (C-1), 130.58 (C-2,6), 159.88 (C-4), 115.60 (C-3,5), 107.00 (C-1’), 160.06 (C-2’), 93.72 (C-3’), 165.87 (C-4’), 95.23 (C-5’), 166.27 (C-6’), 100.63 (C-1"), 73.74 (C-2"), 77.17 (C-3"), 69.94 (C-4"), 77.26 (C-5"), 61.11 (C-6"), 54.85 (OMe).

## Conclusions

Phytochemical study of *Sorbus commixta* fruits led to the isolation of the known chalcone glycoside neosakuranin. This is the first report of the presence of this compound in this species.
